# Inhibition by brimonidine of forskolin-induced nitric oxide synthase expression in human ciliary bodies in vitro

**Published:** 2007-04-02

**Authors:** Renyi Wu, Jinfu Yin, Ke Yao, Ivan Haefliger

**Affiliations:** 1 Eye Center of the 2nd Affiliated Hospital, Medical College of Zhejiang University, Hangzhou, China; 2 Laboratory of Ocular Pharmacology and Physiology, University Eye Clinic Basel, Basel, Switzerland

## Abstract

**Purpose:**

To investigate the mRNA and protein expression of nitric oxide synthase (NOS) in human ciliary bodies in vitro. The effect of the adenylcyclase activator forskolin and/or the α_2_-adrenergic agonist brimonidine (an ocular hypotensive agent that inhibits aqueous humor formation) on NOS mRNA or protein expression was also studied.

**Methods:**

Frozen human ciliary bodies obtained from local eye bank were thawed and incubated with 0.1 mM forskolin for 24 h in the absence or in the presence of 100 μM brimonidine. The mRNA and protein expression of three NOS isoforms (neuronal NOS or nNOS, inducible NOS or iNOS, endothelial NOS or eNOS) were assessed by reverse transcription-polymerase chain reaction (RT-PCR) and Western blot analysis, respectively.

**Results:**

mRNA and protein expression of three NOS isoforms were detected in human ciliary bodies. Forskolin significantly up-regulated the mRNA and protein expression of nNOS, but not that of iNOS or of eNOS. In the presence of brimonidine, the forskolin-induced up-regulation of nNOS mRNA or protein expression was significantly inhibited.

**Conclusions:**

In human ciliary body (where aqueous humor is produced), brimonidine inhibits the up-regulation of nNOS expression induced by forskolin.

## Introduction

Some studies have suggested that nitric oxide (NO) might play a role in the modulation of aqueous humor dynamics in different animal species such as rabbit [1], pig [2], and ox [3]. There exist series of studies supporting the hypothesis that NO may regulate animal or human aqueous humor production. Indeed, in porcine ciliary processes, the site of aqueous humor production, the presence of three NO synthase (NOS) isoforms, namely neuronal NOS (nNOS), or NOS I, macrophagic or inducible NOS (iNOS), or NOS (nNOS), endothelial NOS (eNOS), or NOS III, has been identified by immunostaining [4]. Furthermore, reflecting the biological activity of NOS, NO production could be measured with a porphyrinic NO microsensor [5] applied on the surface of thawed porcine ciliary processes. In similar preparations, when Griess reaction was used to assess nitrite (stable NO metabolite) [6-8], an increased nitrite production was observed when the ciliary processes were exposed to β-adrenergic agonists such as isoproterenol [7] and norepinephrine [8]. In addition, in complement to the observation that β-adrenergic agonists may promote aqueous humor production [9,10], it has been reported that in a guanylate cyclase-cGMP-protein kinase G-dependent manner, NO stimulates stroma-to-aqueous anionic (possibly Cl^-^) transport that therefore might contribute to an increase in aqueous humor formation [11].

In thawed human ciliary processes, NO production could be also detected by a NO microsensor [5]. However, the expressions of the different NOS isoforms have not been illustrated so far in human ciliary processes/body.

The α_2_-adrenergic agonist brimonidine, a potent ocular hypotensive agent, was found to inhibit the nitrite production increase induced by forskolin (an adenylcyclase activator) through an α_2_-adrenoreceptor/Gi-protein pathway activation in porcine ciliary processes [6]. As in porcine ciliary body again, forskolin up-regulates the protein expression of nNOS [12]. However, whether brimonidine modulates NOS expression in animal or human ciliary processes/body is still unclear.

In the present study, we investigated the mRNA and protein expression of three NOS isoforms in human ciliary bodies in vitro. The effect of forskolin and/or brimonidine on NOS expression was also assessed.

## Methods

### Tissue preparation

Human eye tissues were obtained after corneal transplantation from the eye bank of Zhejiang University, Hangzhou, China. Ciliary bodies were dissected free under sterile condition in ice-cold normal saline and stored at -70 °C until enough material was collected to conduct NOS mRNA or protein assessment. Human tissue was handled according to the Declaration of Helsinki and the study protocol was approved by the ethics committee of the 2nd hospital, Medical College of Zhejiang University.

### Experimental procedures

Thawed tissues were washed with Hanks' balanced salt solution (Invitrogen, Beijing, China). Ciliary body rings were cut into halves. One half of each ciliary body ring was used for baseline NOS mRNA or protein determination while the other half was used for a second NOS mRNA or protein assessment after a 24 h exposure to different drugs. For the latter, tissues were placed in a 24-well plate (one half ciliary body ring per well), covered with 1 ml DMEM (Invitrogen) and placed in an incubator (37 °C, 5% CO_2_). Tissues were either left quiescent or incubated with 0.1 μM forskolin and/or 10 μM brimonidine (Sigma-Aldrich, Shanghai, China) for 24 h before another assessment of NOS mRNA or protein expression. Each NOS mRNA or protein determination was made by pooling ciliary body of three to four different eyes.

### Reverse transcription-polymerase chain reaction (RT-PCR) analysis

Total RNA was extracted from tissues using the Trizol Reagent (Invitrogen). cDNA was synthesized according to Reverse Transcription kit manufacturer's instructions (Promega Corp, Madison, WI), and then amplified with a Multiplex polymerase chain reaction (PCR) kit (TaKaRa Corp, Dalian, China). The primers used in this study are presented in Table 1. Cycling parameters for the PCR reactions were as follows: 30 s for annealing at 60 °C, PCR amplification for 30 cycles. Eight μl of the PCR products were analyzed by electro-phoresis on a 1.5% agarose gel. The PCR product was observed using an imaging system (GE Healthcare Life Sciences, Shanghai, China) and the optic density of each band was normalized to the band of β-actin.

**Table 1:** Primers used in RT-PCR analysis of NOS isoforms.

Name	Sequence (5'-3')	Size (bp)
β-actin	Sense: GCCCCTGAGGAGCACCCTGT	
	Antisense: ACGCTCGGTCAGGATCTTCA	300
iNOS	Sense: CCCTTCCGAAGTTTCTGGCAGCAGC	
	Antisense: GGGTGTCAGAGTCTTGTGCCTTTGG	497
eNOS	Sense: CCCTTCCGAAGTTTCTGGCAGCAGC	
	Antisense: GGGTGTCAGAGTCTTGTGCCTTTGG	259
nNOS	Sense: CTGTGACAACTCTCGATACAACATC	
	Antisense: GAGTCTATAGTTGAGCATCTCCTGG	307

### Western blot analysis

Proteins were extracted from the ciliary bodies. Tissues were homogenized and then lysed in a lysis buffer (0.5% Nonidet P-40, 10 mM Tris-HCl (pH 7.5), 150 mM NaCl, 1 mM phenylmethylsulfonyl fluoride and 5 mM aprotinin) for 1 h at 4 °C. The protein concentration in the tissue lysates was determined using the Bio-Rad protein assay kit (Bio-Rad, Shanghai, China). Protein extract (100 ng per lane) was run on a 10% SDS-PAGE gel, and then transferred to a nitrocellulose membrane. The membrane was incubated with anti-human nNOS (1:2000 dilution), iNOS (1:200 dilution), or eNOS (1:1000 dilution) rabbit polyclonal antibodies (Boster Biotechnology Co. Ltd, Wuhan, China). Bound antibody was detected with anti-rabbit immunoglobulin horseradish peroxidase-linked whole antibody and developed by chemiluminescence reaction (Amersham Pharmacia Biotech, Buckinghamshire, UK). The optical density of an entire lane was obtained using Quantity One software (Bio-Rad) and normalized to the band of β-actin.

### Statistical analysis

The data was presented as mean±SD. ANOVA with the Student-Newman-Keuls post hoc analysis was used for the comparison between groups. A p<0.05 was considered statistically significant.

## Results

### Reverse transcription-polymerase chain reaction (RT-PCR) analysis

**Figure 1:**
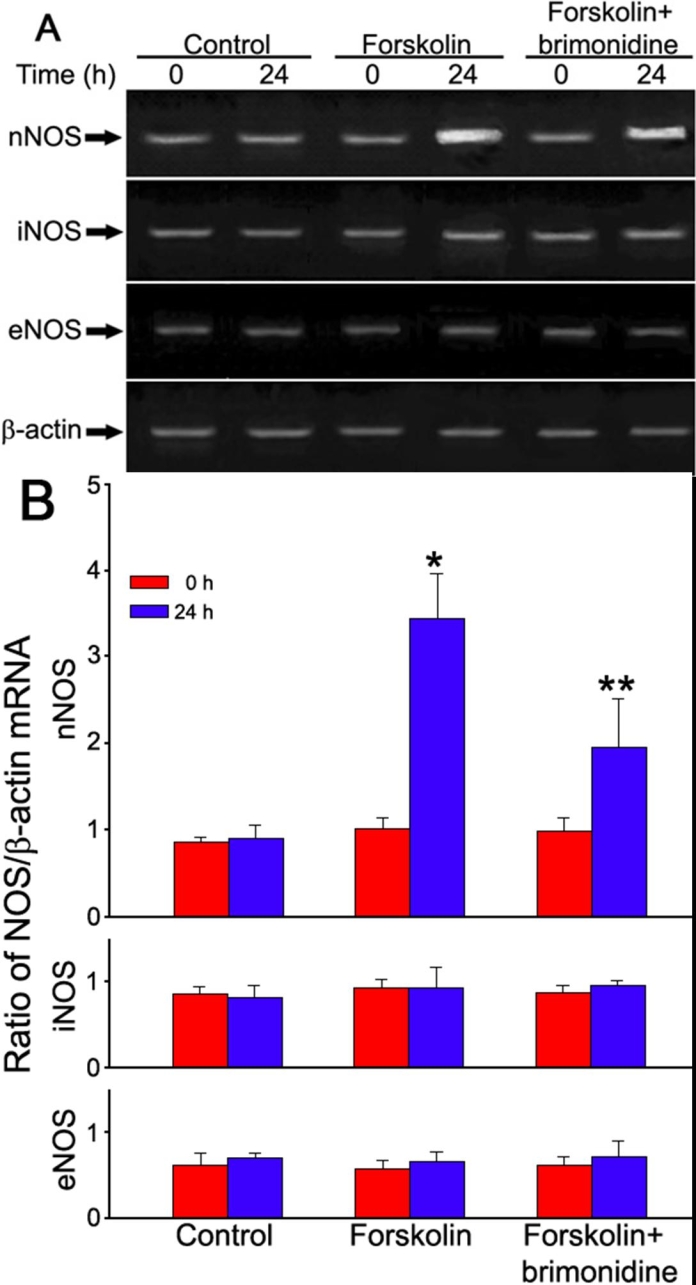
mRNA expression of three NOS isoforms (nNOS, iNOS, and eNOS) in human ciliary bodies. RT-PCR analysis of NOS mRNAs are shown in **A**. Each experiment was repeated 3 times and the mean values of the results of these experiments are shown in **B**. The asterisk indicates a p<0.001, versus forskolin (0 h) and the double asterisk indicates a p<0.05, versus forskolin (24 h).

As shown in Figure 1, mRNA expression of three NOS isoforms was detected in frozen-thawed tissue of human ciliary bodies. After 24 h incubation, significant change in NOS mRNA expression could not be detected either in nNOS, iNOS, or eNOS samples (p>0.05). However, when incubated with 0.1 μM adenocyclase activator forskolin for 24 h, mRNA expression of neuronal NOS (nNOS) was significantly (p<0.001) up-regulated. Brimonidine (10 μM) partially but significantly (p<0.01) inhibited the expression increase of nNOS induced by forskolin. Neither inducible nor endothelial NOS mRNA expression was significantly (p>0.05) influenced by forskolin and/or brimonidine.

### Western blot analysis

**Figure 2:**
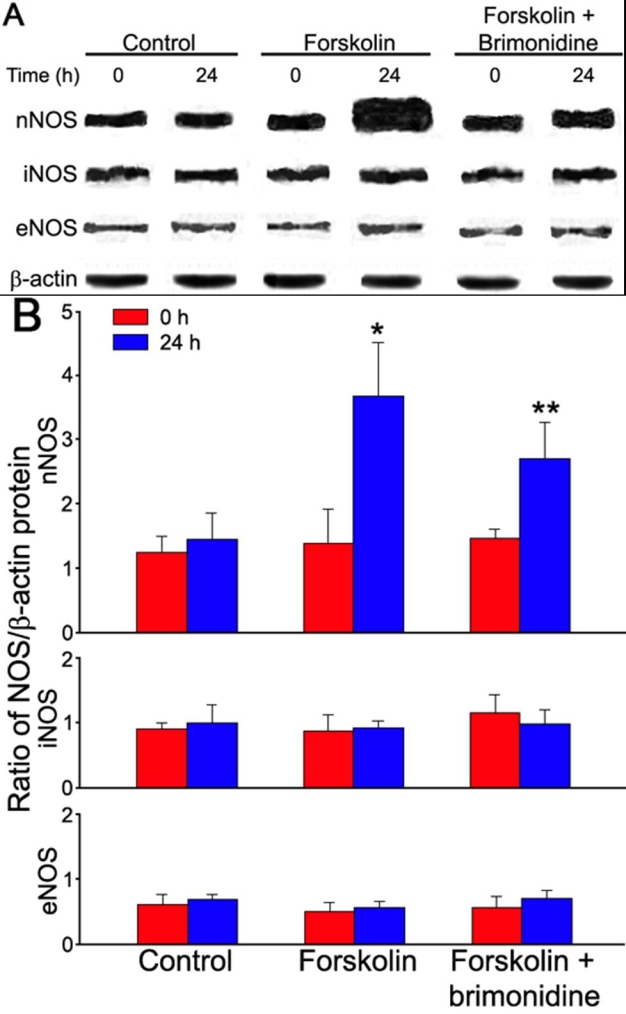
Protein expression of three NOS isoforms (nNOS, iNOS, and eNOS) in human ciliary bodies. Western blot analysis of NOS proteins are shown in **A**. Each experiment was repeated 3 times and the mean values of the results of these experiments are shown in **B**. The asterisk denotes a p<0.01, versus forskolin (0 h) and the double asterisk indicates a p<0.05, versus forskolin (24 h).

To confirm the effect of forskolin and/or brimonidine on NOS isoforms expression, protein products of NOS were analyzed by western blot assay. As shown in Figure 2, Protein expression was detected in frozen-thawed tissues of human ciliary bodies. After 24 h of incubation, significant change in NOS protein expression could not be detected either in nNOS, iNOS, or eNOS samples (p>0.05). The expression of nNOS appeared to be predominant among NOS isoforms. Similar to what was observed in mRNA expression, 0.1 μM forskolin significantly (p<0.01) increase nNOS expression, an increase that was again significantly (p<0.05) attenuated by 10 μM brimonidine. Neither inducible nor endothelial NOS protein expression was significantly (p>0.05) influenced by forskolin and/or brimonidine.

### Nitric oxide synthase expression in freshly harvested human ciliary bodies

Very similar to frozen-thawed tissues, either NOS mRNA or protein expression in freshly harvested human ciliary bodies was up-regulated by 0.1 μM forskolin, and 10 μM brimonidine could again attenuate this up-regulation (data not shown).

## Discussion

The bi-layered epithelium of the ciliary processes/body is known to be the site of aqueous humor production. By immuno-staining, the existence of three NOS isoforms has been seen in porcine ciliary body [4]. In the present study, the mRNA as well as the protein expression of three NOS isoforms was detected in human ciliary body. To our knowledge, this is the first report in the literature about the NOS isoforms mRNA or protein expression in human ciliary body. Although the exact role of nitric oxide production inside the ciliary body is uncertain, the result suggests the possible involvement of NO in physiological and/or pathophysiological processes of the ciliary body, such as aqueous humor production, since NO is highly cell permeable. Indeed, as mentioned before, it has been suggested that NO plays a possible role in aqueous humor production in animal species such as rabbit [1], pig [2], and ox [3]. In pigs, anion (possibly Cl^-^) transport increase is likely to be triggered by NO from the stroma to the aqueous side of the ciliary body [11]. As it is the case in other tissues such as the salivary glands [13], colon [14], and respiratory tract [15], where it has been reported that Cl- transport stimulated by NO is accompanied by fluid secretion, one could speculate that the increase of anion transport in ciliary processes [3] may result in an increase of aqueous humor production. Nevertheless, further investigation is still needed to test the hypothesis that NO might modulate aqueous humor formation in human eyes.

In order to have enough material to conduct experiments (3-4 eyes per experiment), the present study was conducted on human ciliary bodies that underwent a freezing-thawing process. One could therefore argue that this process had, to a certain extent, influenced the nNOS mRNA or protein expression response to the drugs tested. However, in one experiment conducted with a few freshly harvested human ciliary bodies, nNOS mRNA and protein expression also appeared to be up-regulated by 0.1 μM forskolin, an effect that could again be inhibited by 10 μM brimonidine.

Similar to what had been described in porcine ciliary processes [12], in human ciliary body, when exposed to the adenylcyclase activator forskolin, both the mRNA and the protein expression of neuronal NOS, but neither of the inducible NOS nor endothelial NOS, were upregulated. As in porcine ciliary processes, predominant distribution of nNOS was noticed along the junction of the apex-connected epithelial bilayers, suggesting the possibility of nNOS to be involved in the aqueous humor production process of porcine eye [12]. On the other hand, iNOS is always activated by cytokines or endotoxins in some pathophysiological processes such as tissue inflammation [16]. Endothelial NOS is stained in the blood vessel network of the ciliary body and is speculated to be involved mainly in the regulation of ciliary circulation [16]. It has been reported that cAMP may mediate the pharmacological effect of some β-adrenergic agonists to increase aqueous humor production [10]. The result of the present study indicates the upregulation of nNOS expression by forskolin. Taken together, one can speculate that an activation of nNOS is possibly involved in effect of β-adrenergic agonists in the modulation of aqueous humor formation.

As a newly developed hypotensive agent, the mechanism of the α_2_-adrenergic agonist brimonidine on aqueous humor dynamics has not been fully understood. It is reported that through a cAMP-dependent pathway, brimonidine reduces aqueous humor production in human [17] and rabbit [18,19] eyes. The present study showns that in human ciliary bodies, brimonidine significantly inhibited the mRNA and protein expression increase of nNOS induced by forskolin. In one additional experiment, in the absence of forskolin, brimonidine decreased the nNOS mRNA and protein expression slightly (although insignificantly) in human ciliary bodies (data not shown). This insignificance in the nNOS expression inhibition might attribute to either a rather small sample size (n=3), or a relative low level of nNOS expression in normal human ciliary bodies, or both. Nevertheless, the result is in line with the observation made in the porcine ciliary processes, showing that brimonidine prevented the nitrite production increase induced by forskolin. Furthermore, the result again suggested the involvement of NOS, at least nNOS, in the mediation of the pharmacological effect of brimonidine in the eye.

In summary, the present study demonstrated, for the first time, the existence of mRNA and protein expression of three NOS isoforms in human ciliary bodies in vitro. The expression of neuronal NOS was upregulated by the adenylcyclase activator forskolin, an effect that was inhibited by the α_2_-adrenergic agonist brimonidine. The results suggest a possible involvement of NOS in the mediation of the aqueous humor dynamics modulation by brimonidine.
